# P05.14. Educating medical students in clinical perception: an evaluation study

**DOI:** 10.1186/1472-6882-12-S1-P374

**Published:** 2012-06-12

**Authors:** F Edelhäuser, C Scheffer, D Tauschel, M Cosentino, G Lutz, M Neumann

**Affiliations:** 1 The University of Witten/Herdecke, ICURAM, Witten, Germany

## Purpose

Perception is an essential tool used by physicians to develop a comprehensive picture of their patients. A patient-centered approach – in CAM and conventional medicine - puts particular emphasis on perception. Clinical perception techniques are rarely applied or studied in medical and CAM education. The propose of our study was: (1) to illustrate elements of our “Perception Exercise” curriculum and, (2) to conduct a qualitative evaluation of two courses held in 2009 and 2010. The “Perception Exercise” course is part of the patient-centered curriculum at Witten/Herdecke University, which begins in the first year with 6 consecutive practical units of 4 hours.

## Methods

Different perception practices used by our Medical Department over the past 25 years were reevaluated, revised and used in several perception courses. The last two courses were qualitatively evaluated with an anonymous questionnaire composed of five open questions, and an audio-taped group-study of a particular perception exercise, with N=40 participants in total. The evaluations of N=23 participants were analyzed according to Mayring’s content analysis.

## Results

We trained first year medical students in general perception skills using a range of perception and self-awareness exercises and various contact situations with patients. The exercises were primarily based on different didactic approaches such as experienced based education, reflection exercises and self-awareness training. Data analysis showed that the exercises had an emotional and reflective impact on students. This was shown through improved self-awareness that resulted in a more accurate perception and reflection of the patients and patient relationships.

## Conclusion

We have developed a highly interactive seminar in perception exercises that seems to effectively help students understand the importance of perception and self-awareness as an important aspect of accurately perceiving patients’ multifaceted clinical picture. Through the “Perception Exercise” course we aim to cultivate the attitudes and core faculties of the patient-centered physician.

